# Process Intensification at the Nanoscale: Embedding SiC in Zeolites for Energy-Efficient Catalysis

**DOI:** 10.1021/acsomega.4c10598

**Published:** 2025-04-09

**Authors:** Alexandre F. Young, Julia T. de Souza, Antonio M.L.M. Costa, Pedro N. Romano, Javier García-Martínez, João M.A.R. de Almeida

**Affiliations:** † Escola de Química, Universidade Federal do Rio de Janeiro, Av. Athos da Silveira Ramos, 149, Rio de Janeiro 21941-909, Brazil; ‡ Campus Duque de Caxias, Universidade Federal do Rio de Janeiro, Rodovia Washington Luiz, 19593, Rio de Janeiro 25240-005, Brazil; § Laboratorio de Nanotecnología Molecular, Departamento de Química Inorgánica, Universidad de Alicante, 03690 Alicante, Spain; ∥ Instituto de Química, Universidade Federal do Rio de Janeiro, Av. Athos da Silveira Ramos, 149, Rio de Janeiro 21941-909, Brazil; ⊥ Laboratório de Intensificação de Processos e Catálise (LIPCAT), Universidade Federal do Rio de Janeiro, Rua Sydiney Martins Gomes dos Santos, 13 Parque Tecnológico, Cidade Universitária, Rio de Janeiro 21941-859, Brazil

## Abstract

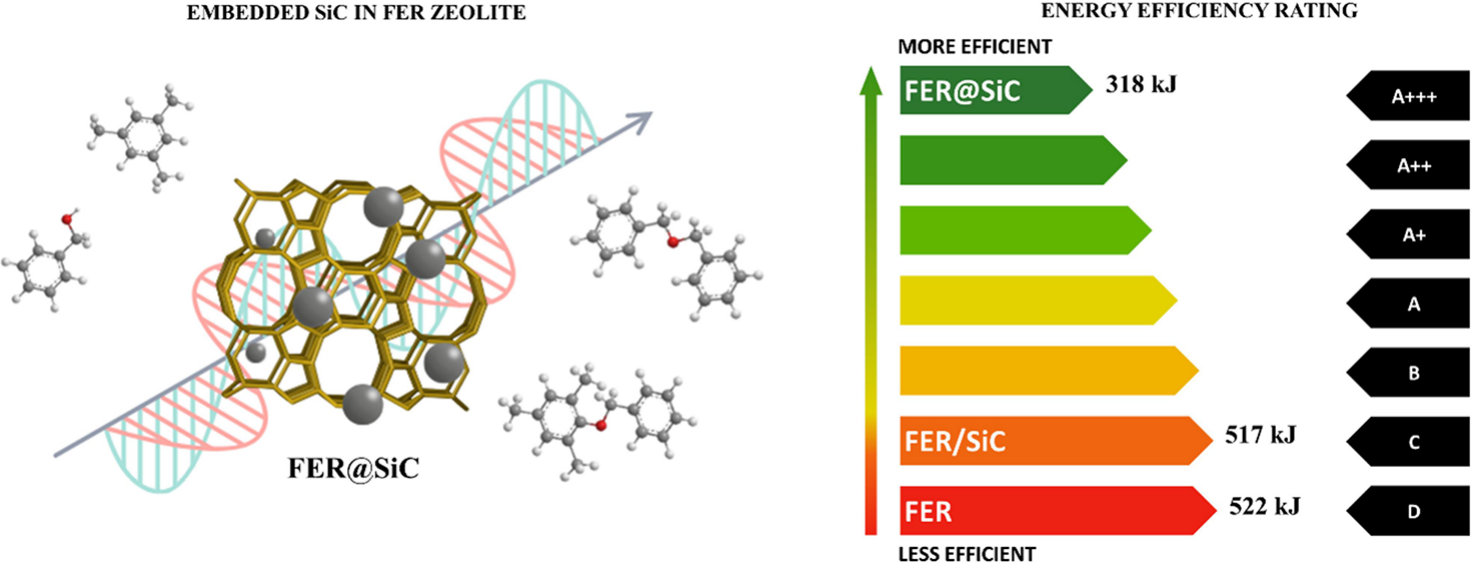

Microwave-absorbent materials are typically blended with low-absorption solids to improve local heating efficiency in catalytic systems. However, the mixing method has a crucial effect on the additive’s heating efficiency. We report here how by embedding silicon carbide (SiC) nanoparticles in ferrierite (FER) zeolite crystals during their synthesis (FER@SiC), a 2.2-fold increase in the catalytic activity for mesitylene and benzyl alcohol alkylation was achieved compared to a physical mixture of FER and SiC nanoparticles (FER/SiC). While the properties of the zeolite in the FER@SiC hybrid and SiC-free FER zeolites are almost identical, we observed a significant increase in catalytic activity under microwave heating when SiC is present within FER crystals. This enhancement is not mirrored by the physical mixture, highlighting the importance of the SiC addition method and the intimate contact between the additive and catalytic phases for effective microwave heating. Actually, FER@SiC achieves the same conversion with 40% less energy, offering insights into designing more efficient zeolite-based catalysts for sustainable chemistry.

## 1 Introduction

Zeolites are extensively utilized microporous materials commonly employed in industrial catalytic processes due to their distinct shape selectivity and acidic characteristics. The use of microwave (MW) irradiation for their synthesis has been thoroughly investigated in the literature.^1−3^ Although the use of MWs on an industrial scale remains a challenge, studies have already reported pilot-scale reactors with capacities of up to 100 L. Such reactors would enable the synthesis of kilograms of catalyst per batch as well as the scale-up of catalytic processes.^4,5^

Most efforts have focused on tuning the synthesis parameters and morphological features to improve the effective heating and thus accelerate the catalytic process. This is particularly important as zeolites, like most aluminosilicates, have a very low MW absorption capacity.^6^ Therefore, high-MW-absorbing additives such as SiC are used with zeolites.^7−10^ Obviously, intimate contact between the two phases is required as this largely determines the effectiveness of the additive in heating the zeolitic catalytic phase. To date, the MW-absorbing material and the zeolite are simply mixed.^11,12^ For example, Fan and co-workers^12^ improved propylene selectivity in methanol-to-hydrocarbon conversion using ZSM-5-coated SiC foams. Similarly, Chaouki and co-workers^13^ applied this to plastics pyrolysis and achieved improved selectivity and conversion, demonstrating the effectiveness of SiC-zeolite hybrids. Conversely, Takanabe and co-workers^11^ improved the proximity between the heating element (in this case, Cs^+^ cations ion-exchanged in an FAU zeolite) and the active sites, achieving remarkable selectivity for catalytic methane combustion under MW heating.

In addition, the MW-absorbing material, such as SiC, can also be used to improve the textural and heat transfer properties of various catalytic processes.^14^ For example, the use of SiC as a framework for zeolite crystallization results in materials with improved diffusion properties. In this context, Alhassan and co-workers^7^ realized this possibility by supporting H–Y zeolite on SiC using a direct hydrothermal method. They observed improved performance in the hexane cracking reaction compared to standard H–Y zeolite and attributed this improvement to the superior textural properties of the SiC-zeolite hybrid.

Expanding on strategies to enhance the MW absorption capacity of advanced catalytic systems, this work presents a new approach to integrating the MW absorber with the catalytic phase. The proposed method involves synthesizing the zeolite in the presence of SiC nanoparticles, leading to a hybrid material where FER plates incorporate SiC nanoparticles both within and between their structures. These solids exhibit much more energy efficiency (40% less energy consumption at constant conversion) and a 2.2-fold increase in catalytic activity in the Friedel–Crafts (FC) process; such a comparison highlights the critical role of SiC addition techniques in optimizing the performance of zeolitic materials for MW-assisted catalytic processes.

## 2 Experimental Section

### 2.1 Catalyst Synthesis

**Figure 1:**
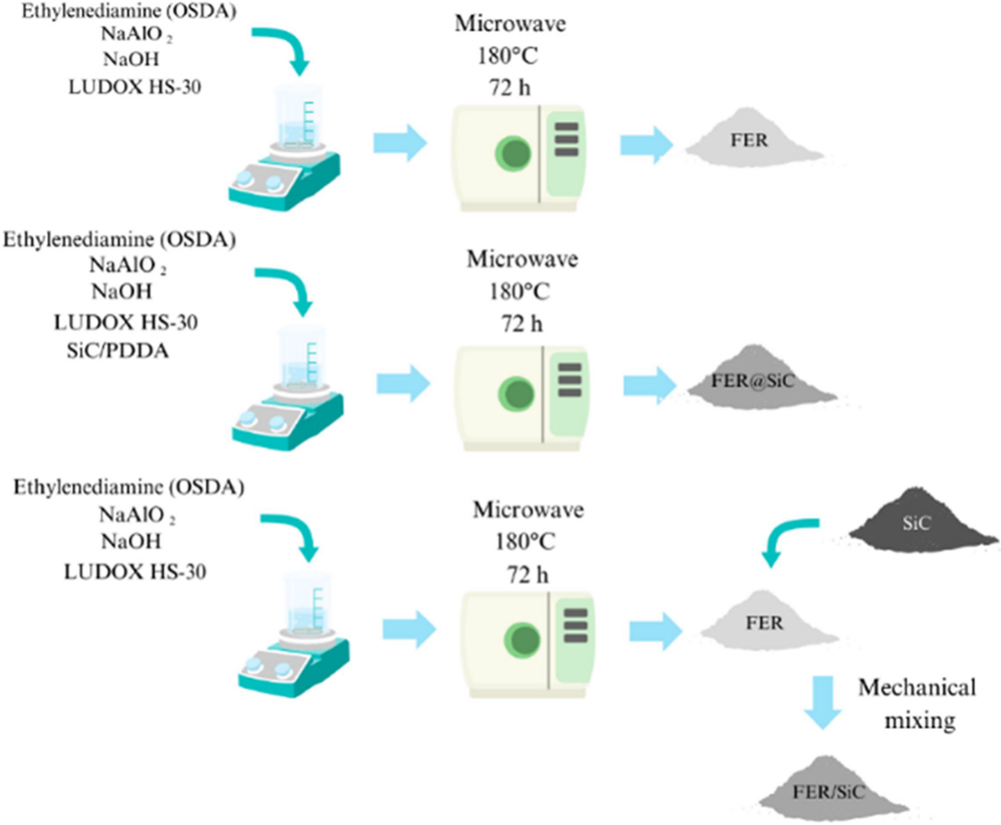
Synthesis schemes for FER, FER@SiC, and FER/SiC.

Ferrierite (FER) zeolite was synthesized following the procedure illustrated in Figure 1, utilizing a hydrothermal method assisted by MW heating. Ethylenediamine (en) served as the organic structure-directing agent (OSDA), while sodium hydroxide, sodium aluminate, and colloidal silica (LUDOX HS-30) provided the necessary sodium, aluminum, and silicon sources, respectively. The synthesis gel, with a molar composition of 19.7 en: 1.85 Na_2_O: 15.2 SiO_2_: 1.0 Al_2_O_3_: 590.0 H_2_O,^15^ was crystallized in an MW reactor at 180 °C for 72 h. Prior to synthesis, 5 g of SiC (nanopowder, < 100 nm particle size, code 594911) was pretreated with 1.25 g of poly(diallyldimethylammonium chloride) (PDDA) in ethanol (25 mL). The solvent was evaporated, and the resulting solid was dried overnight. For FER@SiC, 33% (w/w) of PDDA-modified SiC was incorporated into the synthesis gel before the aging step. The exact amount of pretreated SiC added was determined based on the yield of FER synthesis without SiC. After synthesis, the catalysts underwent calcination at 550 °C for 8 h to remove the OSDA. The acidic form was obtained through ion exchange with 1 M NH_4_Cl at 80 °C for 24 h, followed by a second calcination at 550 °C for 5 h. Additionally, a physical mixture was prepared by maintaining the same mass ratio of SiC to the zeolite phase as in the hybrid material. All reagents were sourced from Sigma-Aldrich.

### 2.2 Characterization

Crystallographic analysis of the materials was performed via X-ray diffraction (XRD) in a Rigaku Miniflex II, operating with CuKα radiation (30 kV and 15 mA). The 2θ angle scanning range was set between 5 and 50 degrees, with an increment of 0.05° every 2 s between each point.

Morphology of the samples was analyzed through scanning electron microscopy (SEM) in a JEOL JSM-IT700HR and through transmission electron microscopy (TEM) using a JEM-1400 Plus microscope (JEOL, 120 kV, 0.38 nm resolution).

The total acidity of the catalysts was evaluated through temperature-programmed desorption of ammonia (NH_3_-TPD) using a Micromeritics AutoChem II Chemisorption Analyzer. Prior to analysis, the samples were pretreated under a helium flow (25 mL/min) at 300 °C for 1 h. Once the system was cooled to 150 °C, ammonia adsorption (15% NH_3_ in He) was conducted for 1 h. To eliminate excess NH_3_, the samples were flushed with helium (50 mL/min), followed by the desorption process, which was performed by heating the reactor to 500 °C at the rate of 10 °C/min.

The textural properties of the materials were characterized by nitrogen adsorption–desorption analysis using an Autosorb iQ (Anton-Paar) instrument. The specific surface area was determined through the BET method, while micropore and mesopore volumes were quantified using NLDFT calculations.

Framework Si/Al of the samples was determined using magic angle spinning nuclear magnetic resonance (MAS NMR) measurements. These were performed on a Bruker Avance III 400WB operating at 79.46 MHz with a single pulse and a rotation rate of 5 kHz for ^29^Si and at 104.23 MHz with a single pulse and a rotation rate of 12 kHz for ^27^Al. Using ^29^Si NMR, it is possible to quantify the number of Si atoms bonded to 0, 1, or 2 Al atoms. On the other hand, ^27^Al NMR allows for the quantification of framework Al atoms and extra-framework Al atoms.

The elemental composition of the samples was determined using an AA-700 atomic absorption spectrophotometer (Shimadzu, Kyoto, Japan). To quantify the aluminum and silicon contents, an MW-assisted acid digestion method was applied. Each 30 mg sample was processed in a Milestone Ethos X Advanced Microwave Extraction system (Metrohm, Herisau, Switzerland) using a reagent mixture consisting of 12 mL of hydrochloric acid (HCl), 4 mL of nitric acid (HNO_3_), and 1 mL of hydrofluoric acid. The digestion process was carried out in two stages: an initial heating to 170 °C for 5 min, followed by an increase to 200 °C for 25 min, with a total duration of 60 min under controlled MW power. After digestion, the samples were cooled to room temperature, and the acidic solution was neutralized with 6 mL of boric acid. The final solution was filtered to remove any residual particulates and diluted with ultrapure water before analysis.

The alkylation products were analyzed qualitatively using a GC-MS QP2020 NX (Shimadzu) with a DB-1-MS column, while the quantification was carried out using a GC-FID 2030 (Shimadzu) with a DB-1 column, based on the relative areas of the chromatographic peaks.

For the GC-FID method, the initial oven temperature was set to 35 °C, followed by an increase to 240 °C at a rate of 10 °C/min, where it was held for 10 min. Then, the temperature was raised to 320 °C at the same rate and held for an additional 0.5 min. Helium was used as the carrier gas with a constant pressure of 248 kPa. The injector temperature was maintained at 275 °C, and the detector was set to 320 °C.

### 2.3 Catalytic Evaluation

In the FC alkylation reaction involving mesitylene and benzyl alcohol (BA), 95 mmol of mesitylene, 1 mmol of BA, and 100 mg of zeolite were used for each experiment. The reaction was carried out at 120 °C with continuous stirring at 300 rpm, using either conventional heating (CH) or MW-assisted heating. Reactions carried out under CH were conducted in round-bottomed flasks in an oil bath. MW-assisted reactions were carried out in a CEM Discover 2.0 reactor. All reactions were performed in triplicate to obtain error data. In the CH setup, a round-bottom flask system containing mesitylene and the catalyst in an oil bath was heated to 120 °C, after which BA (the limiting reagent) was added. A sample was taken after heating, and no product formation was observed until the addition of BA. In the MW reactor, due to equipment limitations, the closed reaction system containing all reagents and the catalyst was heated to 120 °C. A sample was collected as soon as the final temperature was reached, showing a conversion of less than 1%. Thus, the heating time was considered negligible. A reaction experiment was conducted for each MW reaction time and each catalyst. In order to evaluate the conversion of BA and the selectivity for 1,3,5-trimethyl-2-benzylbenzene (TM2B) and dibenzyl ether (DBE), GC-FID analysis was performed.^16^ The energy spent in each test in kJ for MW heating was calculated by integrating the instantaneous power measurement from the MW reactor with reaction time.

### 2.4 Temperature Calibration for Catalytic Evaluation

To ensure reliability in the catalytic evaluation data, a vessel was heated in an oil bath until it reached a temperature of 100 °C. The temperature was monitored using a calibrated thermometer to ensure accuracy. Subsequently, the vessel was transferred to the CEM Discover 2.0 reactor, and the temperature, measured by an infrared detector and displayed on the equipment, was observed. It was found that the measured temperature was the same as the temperature recorded by the thermometer during the oil bath heating process, indicating consistency in temperature measurement across the different setups.

## 3 Results and Discussion

**Figure 2:**
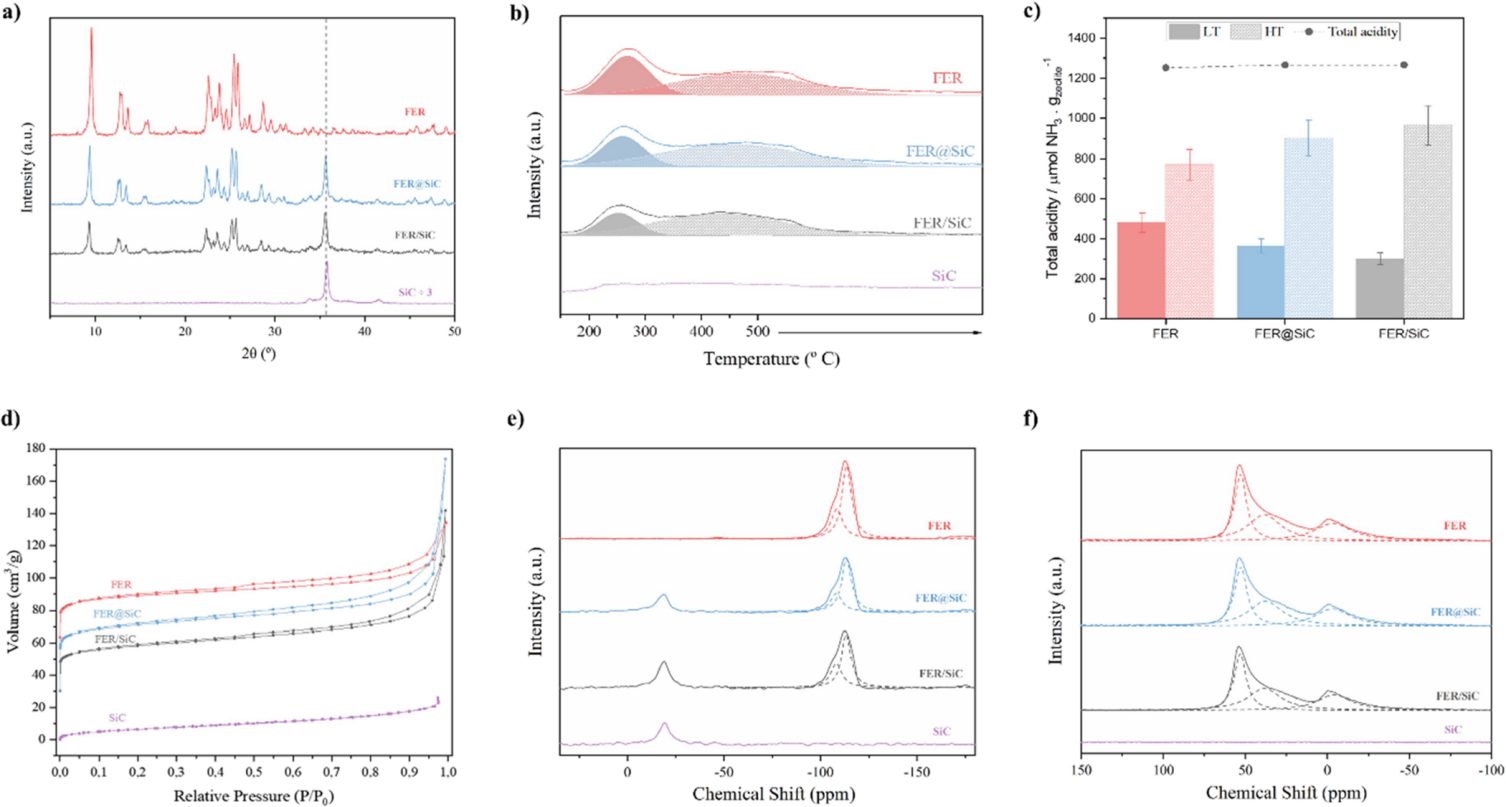
Characterizations of the catalysts. (a) XRD patterns of the FER, FER@SiC, FER/SiC, and SiC samples. (b) NH_3_-TPD of the FER, FER@SiC, FER/SiC, and SiC samples. (c) Total acidity per gram of zeolite for FER, FER@SiC, and FER/SiC samples. (d) N_2_ physisorption isotherms at 77 K of FER, FER@SiC, FER/SiC, and SiC. (e) ^29^Si NMR for FER, FER@SiC, FER/SiC, and SiC samples. (f) ^27^Al NMR for FER, FER@SiC, FER/SiC, and SiC samples.

All zeolite-based catalysts utilized in this study consist of highly crystalline FER, as confirmed by XRD analysis (Figure 2a). No additional crystalline phases were detected, except for the SiC that was incorporated. The hybrid material (FER@SiC), prepared under the same conditions as the pure FER sample but in the presence of SiC nanoparticles, shows the same XRD pattern in addition to the characteristic diffraction peaks attributable to SiC. The reduction in intensity is due to the lower (one-third) amount of zeolite in the hybrid and is similar to that observed for the physical mixture FER/SiC.

The acidic properties of the samples were determined by NH_3_-TPD (Table 1 and Figure 2b), further confirming that the main properties of the zeolite, in this case, the total acidity, are not affected by the presence of SiC. When compared at a constant zeolite content (Figure 2c), the acidity profiles of the materials show no significant differences that could be attributed to the presence of SiC.

**Table 1:** Acidic and Textural Properties of SiC and the Synthesized Materials

catalyst	*S*_BET_ (m^2^/g_cat_)	*S*_BET_ (m^2^/g_zeolite_)	*V*_mic_^a^ (cm^3^/g_cat_)	*V*_mic_^a^ (cm^3^/g_zeolite_)	*V*_meso_^a^ (cm^3^/g_cat_)	total acidity (μmol/g_zeolite_)	bulk Si/Al^b^	framework Si/Al^c^
FER	356	356	0.14	0.14	0.02	1254	8.2	10.7
FER@SiC	277	415	0.09	0.14	0.04	1268	12.9	10.9
FER/SiC	225	337	0.09	0.14	0.04	1349	14.5	10.1
SiC	30		<0.01		0.04	190		

a Micro- and mesopore volumes calculated through NLDFT.

b Bulk Si/Al obtained through atomic absorption spectrometry.

c Framework Si/Al obtained through ^29^Si and ^27^Al NMR.

Textural characterization of the materials (Table 1 and Figure 2d) shows that the textural properties of FER and FER@SiC are comparable when normalized by the amount of zeolite in the hybrid (33 wt %), excluding any significant pore blocking due to the presence of SiC in the FER@SiC sample. When the micropore volume is normalized by the zeolite mass, typical FER topology volumes are observed for both the hybrid material and the physical mixture, which present similar values.^17^

**Figure 3:**
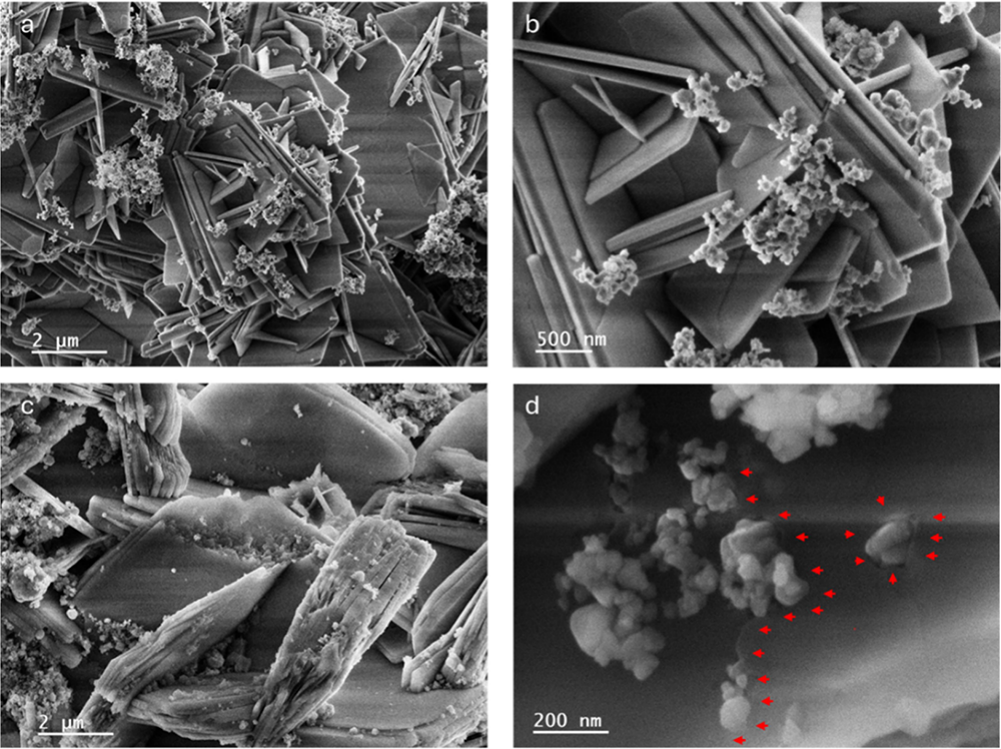
Secondary electron SEM micrographs of (a) and (b) physical mixture FER/SiC and (c) and (d) hybrid FER@SiC.

**Figure 4:**
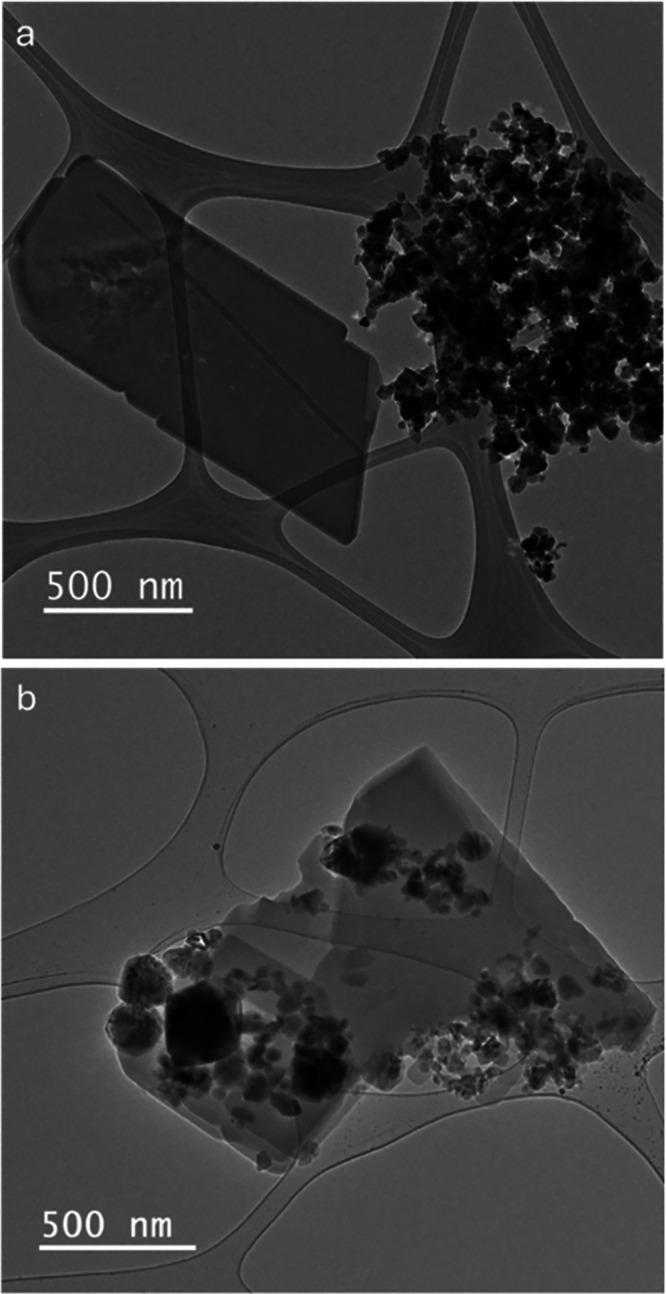
Bright-field TEM of (a) physical mixture FER/SiC and (b) hybrid FER@SiC.

Figure 3a,b reveals the characteristic plate-like morphology of FER zeolite mixed with SiC nanoparticles, which exhibit poor contact with each other. In Figure 3b, we observe that the FER plates are well-defined and faceted. Interestingly, the hybrid FER@SiC images (Figure 3c,d) indicate that the growth of plate-like FER zeolite is partially obstructed by the addition of SiC nanoparticles. These nanoparticles attach to the growing FER surface, hindering the continuous plate growth, which results in irregular edges (Figure 3c) and the embedding of SiC nanoparticles during FER growth, as indicated by the red arrows (Figure 3d). These observations are corroborated by the TEM analyses shown in Figure 4. Figure 4a illustrates the isolated FER zeolite plates and SiC nanoparticles with minimal physical contact in the physical mixture, while FER@SiC (Figure 4b) displays SiC particles attached to, and embedded within, the zeolite, like the one depicted in Figure 3d.^11^

As will be demonstrated later, this difference significantly impacts the catalytic performance of the materials under MW heating. The micrographs shown in Figures 2 and 3 are representative examples of an extensive study conducted to gain a comprehensive understanding of the morphology of the catalysts and the location of the SiC. For additional micrographs, the reader is referred to Figures S1–S7.

The elemental analysis of the materials was conducted using atomic absorption spectroscopy (Table 1). Interestingly, the Si/Al ratio of the hybrid catalyst is one-third higher than that of FER, indicating that the only chemical change between the materials is the presence of added SiC.

Furthermore, the structural analysis using ^29^Si and ^27^Al NMR (Figure 2e,f and Table 1) shows that the framework Si/Al ratios for both FER and FER@SiC remain identical, which supports the observation that the addition of SiC does not alter the zeolitic structure. This conclusion is further reinforced by Figure 2e, which demonstrates no change in the silicon (Si) bonds, as indicated by the consistent peak intensities observed between the hybrid material and the physical mixture.

**Figure 5:**
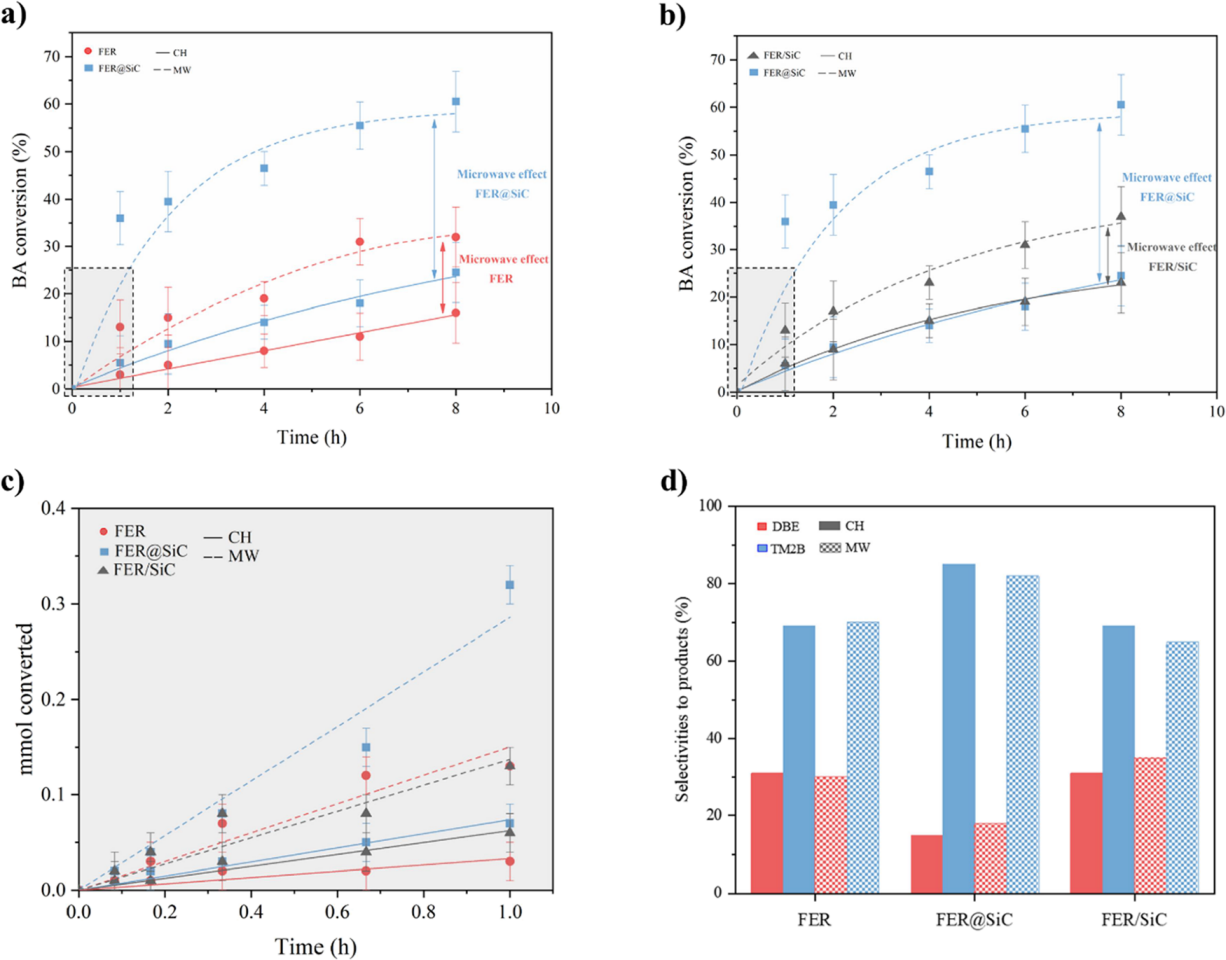
BA conversion over different reaction times for (a) FER and FER@SiC, (b) FER/SiC, FER@SiC, and (c) initial BA conversion rates for all three materials. (d) Selectivities toward DBE and TM2B under both CH (solid) and MW heating (lines) for FER, FER@SiC, and FER/SiC at an isoconversion of 15%. Zeolite mass was maintained constant for all catalytic tests.

The structural, morphological, and acidity characterizations of the samples show that the zeolite in the FER@SiC hybrid and in the FER/SiC physical mixtures is basically identical to the pure FER phase. However, a striking difference in catalytic activity is observed in the FC alkylation between mesitylene and BA, especially under MW irradiation (Figure 5). In Figure 5, panels a and b present the catalytic activities of the three materials. In Figure 5a, the zeolite is compared with the embedded catalyst, while in Figure 5b, FER@SiC is compared with the physical mixture. Both figures feature a shaded square highlighting the region of low activity. The initial data points were omitted in these figures to avoid compromising the clarity of the graph. However, Figure 5c specifically displays the activities of the three materials in this low-activity region.

Figure 5a shows the conversion rates of FER and FER@SiC under both CH and MW heating. Under CH, both materials show similar conversion rates, with FER@SiC showing slightly better performance. However, under MW irradiation, FER@SiC shows a remarkable increase in catalytic activity compared to FER, as expected due to the excellent MW-absorbing properties of ceramic materials like SiC.^18^

More interestingly, FER@SiC and FER/SiC, which behave almost identically under CH, are significantly different under MW irradiation (Figure 5b). The intimate contact between the SiC and the FER, obtained by growing the zeolite crystals in the presence of SiC nanoparticles, results in a 2.2-fold improvement over the physical mixture after the first hour of reaction, in which FER/SiC leads to a conversion of 0.14 mmol of BA, while FER@SiC presented a conversion of 0.32 mmol (Figure 5c).

This result, corroborated by the higher initial reaction rate of FER@SiC, clearly demonstrates the importance of the intimate contact between the heating and catalyst phases and shows the potential of the technique we disclose here.^14^

Interestingly, and despite the significant increase in activity observed when the SiC and FER were in intimate contact (FER@SiC), there were no observable changes in selectivity for any of the systems under either CH or MW heating (Figure 5d). The slight improvement in the production of the TM2B (alkylation product) for FER@SiC could be due to a combination of micro- and mesoporous structures that improve selectivity.^19^ These results highlight once again that the intrinsic properties of the zeolite, which determine its selectivity, are basically unaffected by the presence of SiC, which only acts as a heating element without significantly altering the intrinsic catalytic properties of the zeolite.

**Figure 6:**
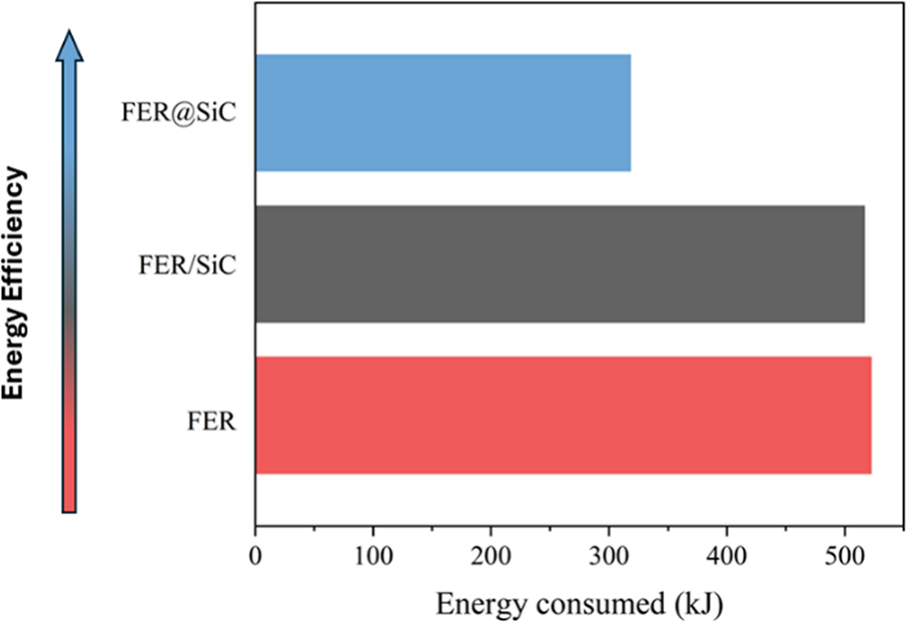
Energy consumption in the MW reactor for each catalyst at a BA isoconversion of 15%.

The remarkable role played by the intimate contact between the heating element (SiC) and the catalytic phase (FER) is well illustrated in Figure 6, which shows the energy consumption required to achieve 15% conversion. These data were obtained from the MW reactor, considering the total power consumed by each material up to the final time point, where the isoconversion is observed. It is noteworthy that FER@SiC requires only about 60% of the energy input of either FER or FER/SiC. This result further confirms that simply blending high MW-absorbing materials is not a very effective way to improve the local heating efficiency due to poor contact. The significant improvement in the catalytic performance of FER@SiC over FER/SiC is attributed to the close contact between the MW absorber, i.e., the SiC nanoparticles, and the catalytic phase, i.e., the FER zeolite (see Figure 3), since other important parameters such as porosity, crystal morphology, acidity, and Si/Al (Table 1) are almost identical.

## 4 Conclusions

By embedding SiC nanoparticles into FER zeolite crystals during synthesis, we achieved a significant enhancement in catalytic performance for the FC alkylation of mesitylene with BA under MW heating, surpassing the activities of both the SiC-free system and the physical mixture.

Our results highlight the essential influence of the close interaction between the heating medium (SiC) and the catalytic phase (FER zeolite) in facilitating effective MW-assisted catalysis, as evidenced by the superior conversion rate, close to 2.2-fold, and a remarkable reduction in energy consumption, exceeding 40%, when comparing the FER zeolites with the SiC nanoparticles inside and outside. These results open new opportunities for the rational design of more efficient zeolite-based catalysts, thus advancing the field of MW chemistry toward greater attractiveness and practicality.

## Supporting Information Available

Other SEM images of the materials, including SEM-EDS images (PDF)
