# Supplementary material for: Process Intensification at the Nanoscale: Embedding SiC in Zeolites for Energy-Efficient Catalysis
Source: ACS Omega. 2025 Apr 9;10(15):15075–81. doi: 10.1021/acsomega.4c10598 (PMC12019495; doi:10.1021/acsomega.4c10598)
Supplement: Supplementary file 1 — ao4c10598_si_001.pdf [file ao4c10598_si_001.pdf]

Supporting Information

Process Intensification at the Nanoscale: Embedding SiC in Zeolites for Energy-Efficient Catalysis

Alexandre F. Young[a,e], Julia T. de Souza[a,e], Antonio M.L.M. Costa[e], Pedro N. Romano[b,e], Javier García-Martínez[c,] and João M.A.R. de Almeida[d,e,*]*

[a] Escola de Química, Universidade Federal do Rio de Janeiro, Av. Athos da Silveira Ramos, 149, Rio de Janeiro, Brazil, 21941-909.

[b] Campus Duque de Caxias, Universidade Federal do Rio de Janeiro, Rodovia Washington Luiz, 19593, Rio de Janeiro, Brazil, 25240-005.

[c] Laboratorio de Nanotecnología Molecular, Departamento de Química Inorgánica, Universidad de Alicante, 03690, Alicante, Spain

[d] Instituto de Química, Universidade Federal do Rio de Janeiro, Av. Athos da Silveira Ramos, 149, Rio de Janeiro, Brazil, 21941-909.

[e] Laboratório de Intensificação de Processos e Catálise (LIPCAT), Universidade Federal do Rio de Janeiro, Rua Sydiney Martins Gomes dos Santos, 13 Parque Tecnológico, Cidade Universitária, Rio de Janeiro, Brazil, 21941-859.

*j.garcia@ua.es (Javier García-Martínez)
*j.monnerat@iq.ufrj.br (João M.A.R. de Almeida)

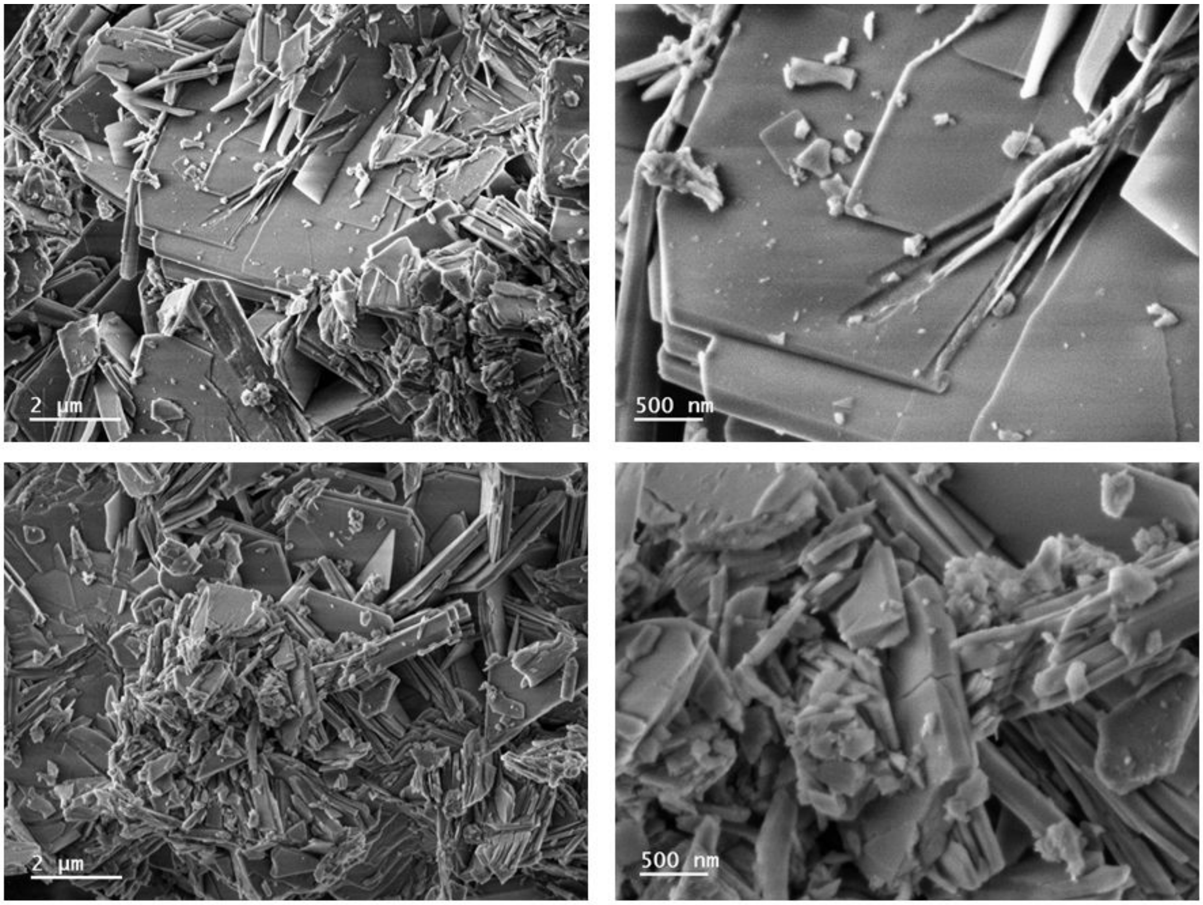

Figure S1. SEM imagens of FER.

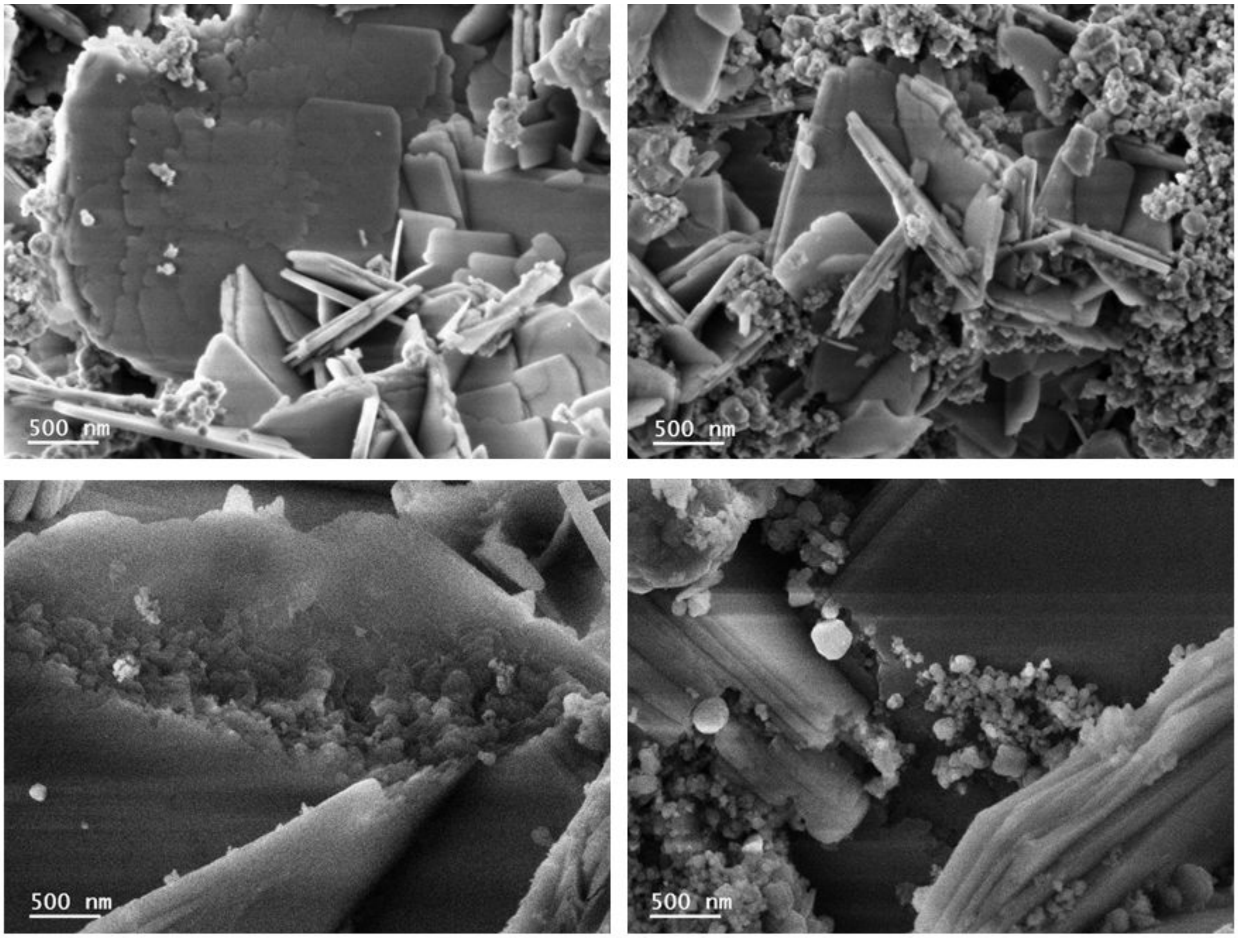

Figure S2. SEM imagens of FER@SiC.

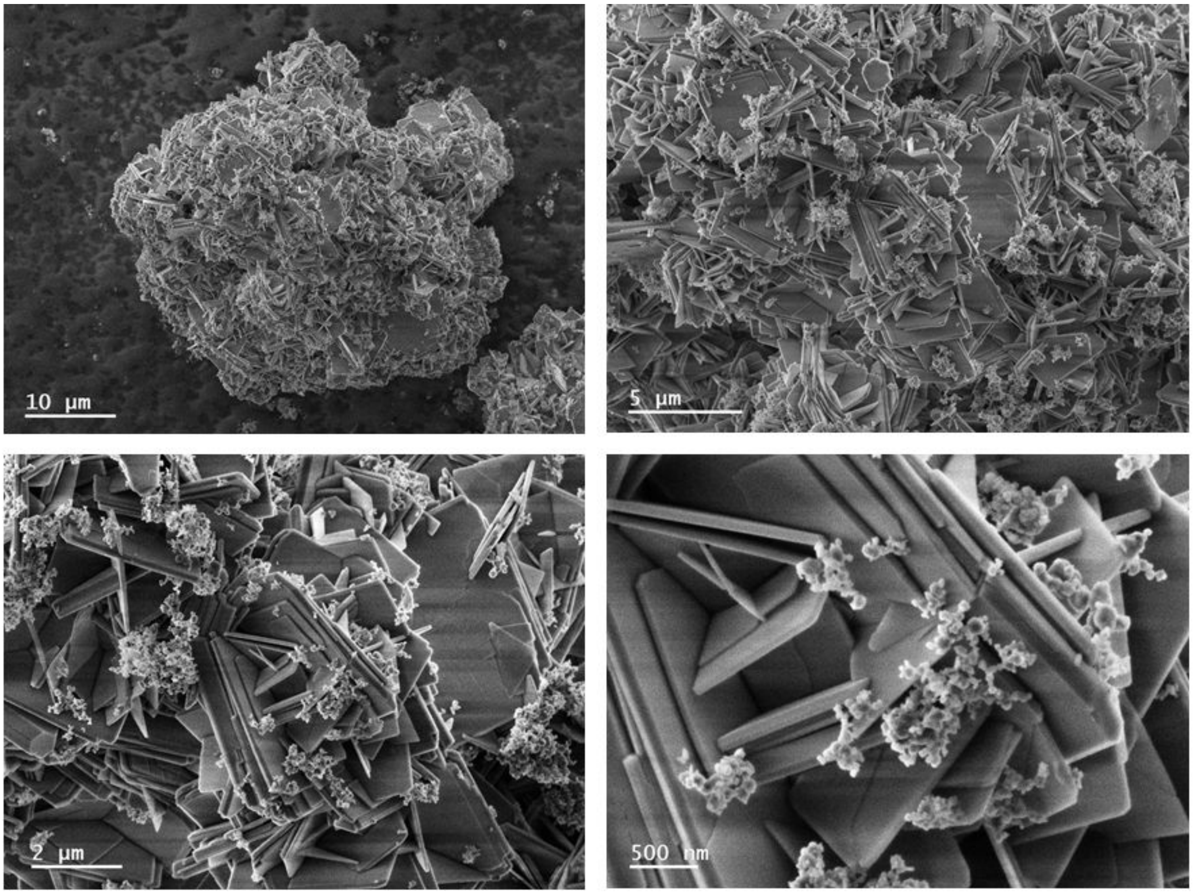

Figure S3. SEM imagens of FER/SiC.

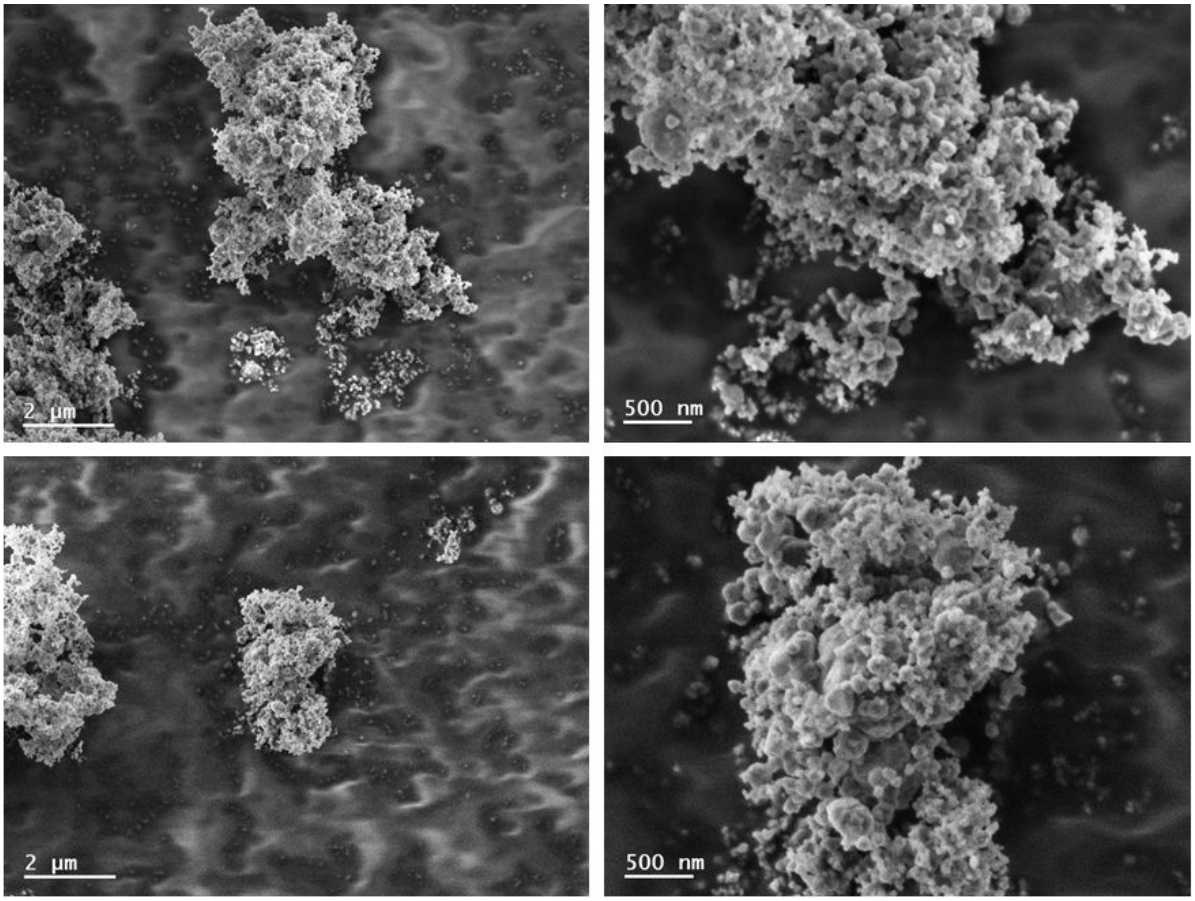

Figure S4. SEM imagens of SiC.

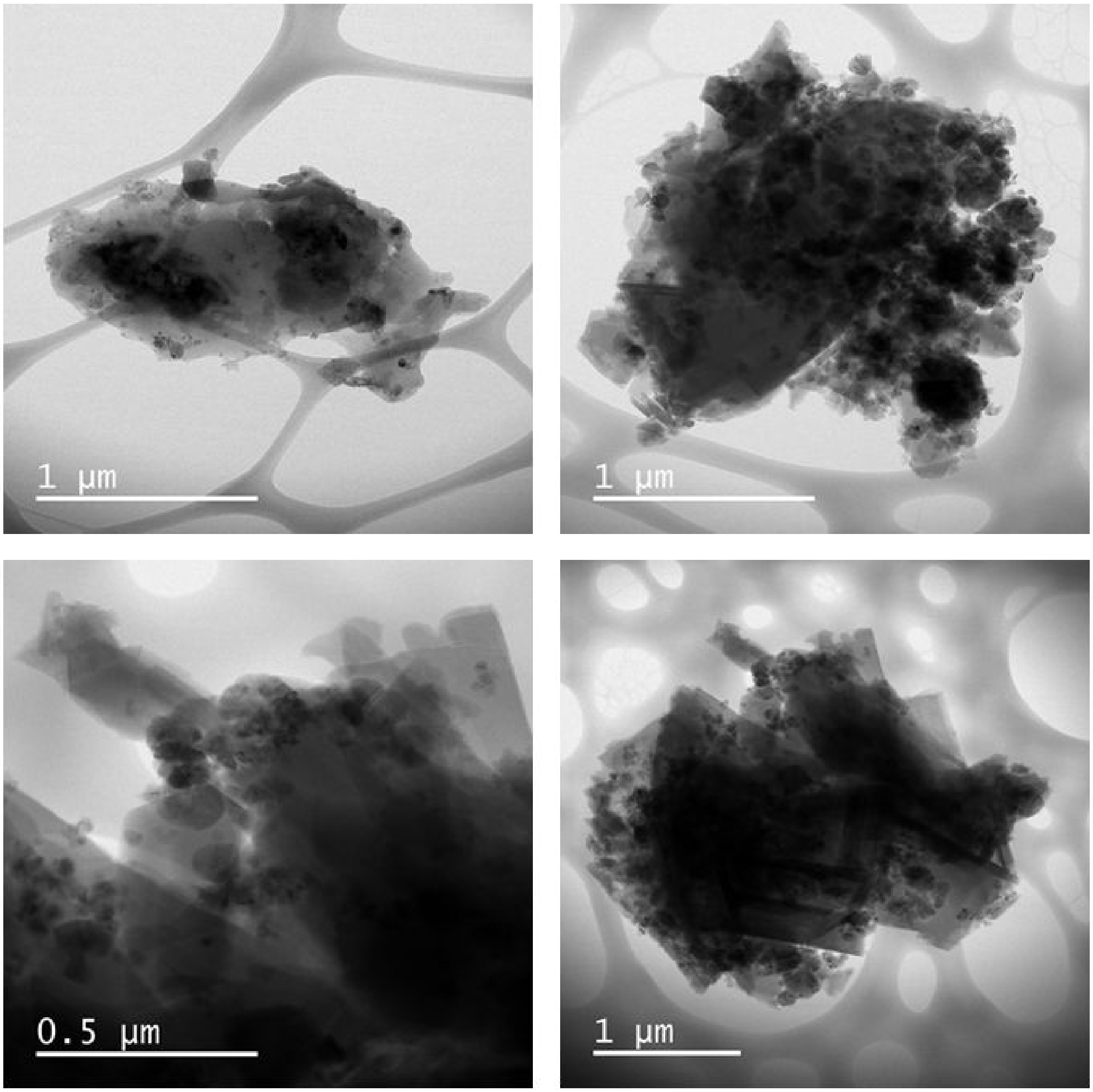

Figure S5. TEM imagens of FER@SiC.

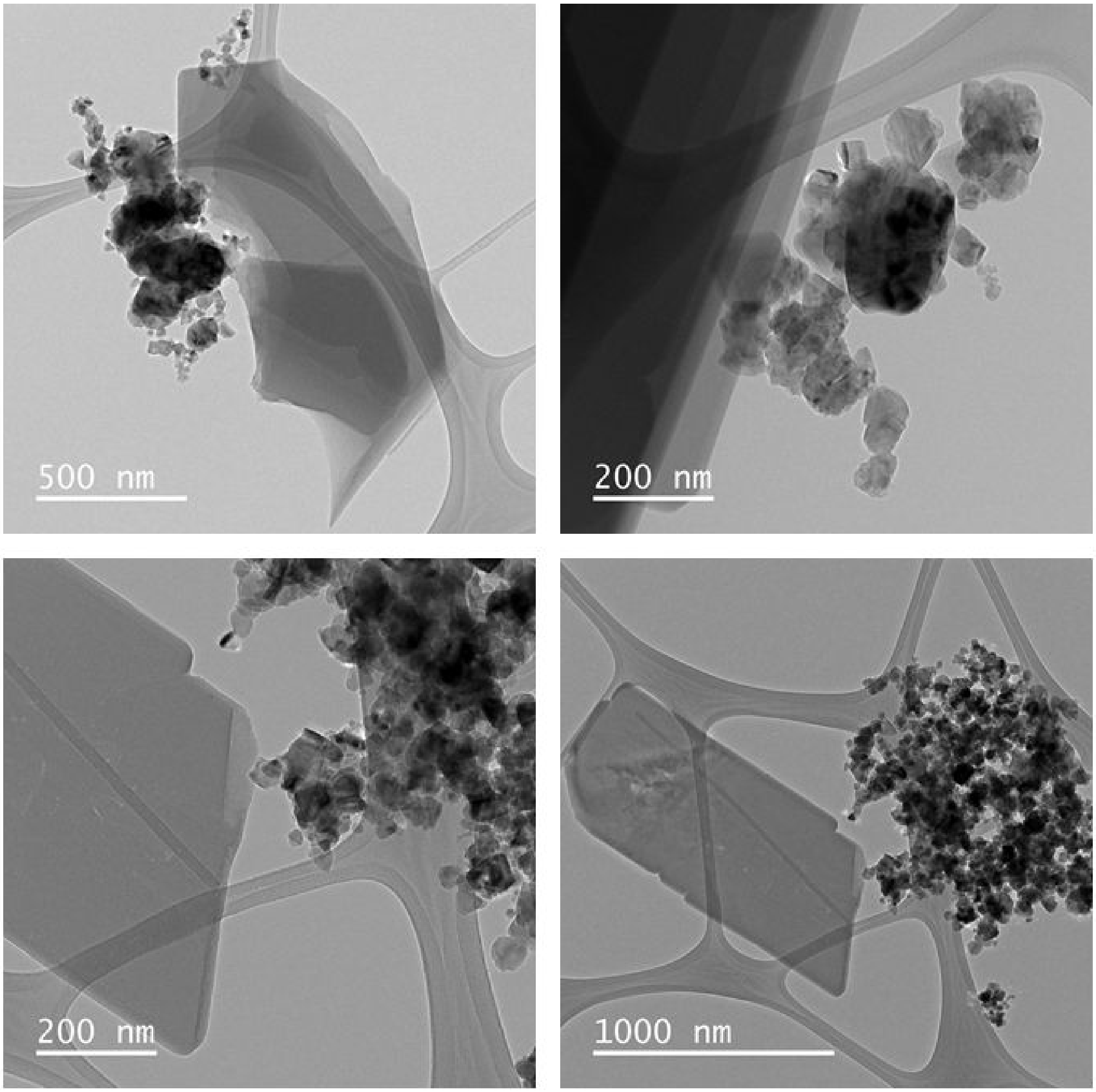

Figure S6. TEM imagens of FER/SiC.

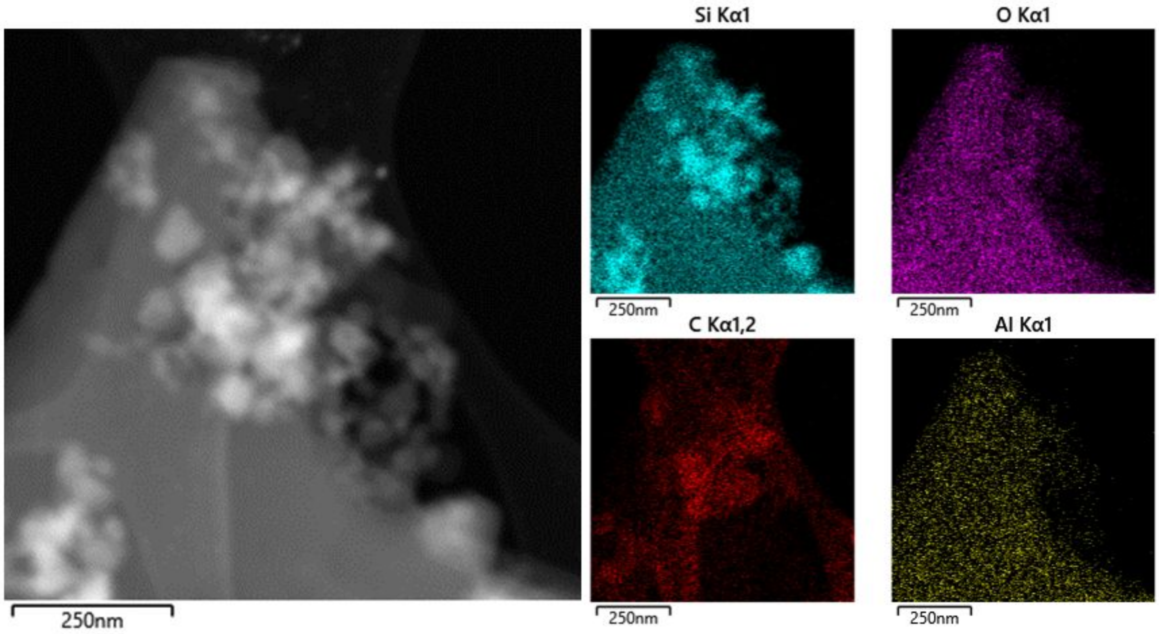

Figure S7. HAADF/STEM and EDS mapping imagens of FER@SiC.
